# The peripheral chimerism of bone marrow–derived stem cells after transplantation: regeneration of gastrointestinal tissues in lethally irradiated mice

**DOI:** 10.1111/jcmm.12227

**Published:** 2014-01-20

**Authors:** Stanislav Filip, Jaroslav Mokrý, Jiřina Vávrová, Zuzana Šinkorová, Stanislav Mičuda, Pavel Šponer, Alžběta Filipová, Hana Hrebíková, Govindan Dayanithi

**Affiliations:** a Department of Oncology and Radiotherapy, Faculty of Medicine and Teaching Hospital, Charles University in PragueHradec Králové, Czech Republic; b Department of Histology and Embryology, Faculty of Medicine, Charles University in PragueHradec Králové, Czech Republic; c Department of Radiobiology, Faculty of Military Health Science, University of DefenceHradec Králové, Czech Republic; d Department of Pharmacology, Faculty of Medicine, Charles University in PragueHradec Králové, Czech Republic; e Department of Orthopaedics, Faculty of Medicine, Charles University in PragueHradec Králové, Czech Republic; f Department of Medical Biochemistry, Faculty of Medicine, Charles University in PragueHradec Králové, Czech Republic; g Department of Molecular Neurophysiology, Institute of Experimental Medicine, Academy of SciencePrague, Czech Republic; h Institut National de la Santé et de la Recherche Médicale, Unité de recherche U710, Université Montpellier 2Montpellier Cedex 5, France; i Ecole Pratique des Hautes EtudesParis, France

**Keywords:** Chimerism, cell recruitment, cell trafficking, stem cells, tissue regeneration

## Abstract

Bone marrow–derived cells represent a heterogeneous cell population containing haematopoietic stem and progenitor cells. These cells have been identified as potential candidates for use in cell therapy for the regeneration of damaged tissues caused by trauma, degenerative diseases, ischaemia and inflammation or cancer treatment. In our study, we examined a model using whole-body irradiation and the transplantation of bone marrow (BM) or haematopoietic stem cells (HSCs) to study the repair of haematopoiesis, extramedullary haematopoiesis and the migration of green fluorescent protein (GFP^+^) transplanted cells into non-haematopoietic tissues. We investigated the repair of damage to the BM, peripheral blood, spleen and thymus and assessed the ability of this treatment to induce the entry of BM cells or GFP^+^lin^−^Sca-1^+^ cells into non-haematopoietic tissues. The transplantation of BM cells or GFP^+^lin^−^Sca-1^+^ cells from GFP transgenic mice successfully repopulated haematopoiesis and the haematopoietic niche in haematopoietic tissues, specifically the BM, spleen and thymus. The transplanted GFP^+^ cells also entered the gastrointestinal tract (GIT) following whole-body irradiation. Our results demonstrate that whole-body irradiation does not significantly alter the integrity of tissues such as those in the small intestine and liver. Whole-body irradiation also induced myeloablation and chimerism in tissues, and induced the entry of transplanted cells into the small intestine and liver. This result demonstrates that grafted BM cells or GFP^+^lin^−^Sca-1^+^ cells are not transient in the GIT. Thus, these transplanted cells could be used for the long-term treatment of various pathologies or as a one-time treatment option if myeloablation-induced chimerism alone is not sufficient to induce the entry of transplanted cells into non-haematopoietic tissues.

## Introduction

Stem cells (SCs) and progenitor cells play an important role in regenerative medicine. The main considerations in using transplanted cells are the availability and suitability of resources for the uncomplicated collection of cells for transplant, the regenerative potential of the transplanted cells and the appropriate application of these cells [1]. Within the haematopoietic system, SCs and progenitor cells meet most of the above-mentioned criteria and, as such, are the main types of cells that are used in regenerative medicine [2,3]. The therapeutic benefit of haematopoietic stem cells (HSCs) has been demonstrated in numerous clinical and experimental studies, and they have been shown to be effective for a broad range of conditions [4–6].

The main goal of regenerative medicine is to understand the mechanisms by which the transplantation of cells with high regenerative potential leads to the regeneration of damaged tissues. These mechanisms are based on cell plasticity and cell differentiation, cell fusion or paracrine mechanisms [7]. The application of SCs in treatment is associated with a low efficiency of homing and attachment to the injury site [8]. Our experimental work is focused on monitoring the circulation of transplanted cells and their homing properties, as well as the involvement of endogenous cells in the process of repairing damaged tissues. To model the treatment of acute radiation syndrome (ARS), we used a model of bone marrow cell transplantation using full bone marrow transplantation (BMT) or separated HSCs from the bone marrow [9]. In our early experiments using this model, we found that bone marrow cells and cells separated from the bone marrow that were homed to extramedullary tissues with different kinetics [5,7].

Chimerism is the process by which injected bone marrow cells from a donor repopulate the haematopoietic system of a recipient. The study of chimerism was facilitated by intravenous tracking of sorted green fluorescent protein (GFP^+^) or GFP^+^lin^−^Sca-1^+^ bone marrow cells in a GFP^−^ background. Numerous models have been designed to induce chimerism in mouse models, such as parabiosis, immunosuppression of the recipient mice and whole-body irradiation [10]. It appears that only lethal doses of whole-body irradiation are able to efficiently create chimerism and to induce the migration of GFP^+^ bone marrow cells or GFP^+^lin^−^Sca-1^+^ cells to the gastrointestinal tract. Concerns have arisen regarding the use of irradiation in a clinical sitting as the effects of this treatment on intestinal integrity and on the permeability of the blood–intestinal barrier are poorly understood. A model of whole-body irradiation in which the intestines were protected from irradiation showed that the transplanted cells could not infiltrate tissues, such as the small intestine and liver. Thus, this suggested that irradiation-induced damage to the intestine was responsible for the entry of transplanted cells into the small intestine and liver. Following whole-body irradiation in which the body was protected, there was a lack of invasion of bone marrow or lin^−^CD117^+^ transplanted cells. This demonstrated that haematopoietic stem cell transplantation not only led to the recovery of haematopoietic and lymphoid tissues but also facilitated the recovery of the small intestine mucosa, which was significantly damaged by ionizing radiation [11,12].

**Fig. 1:**
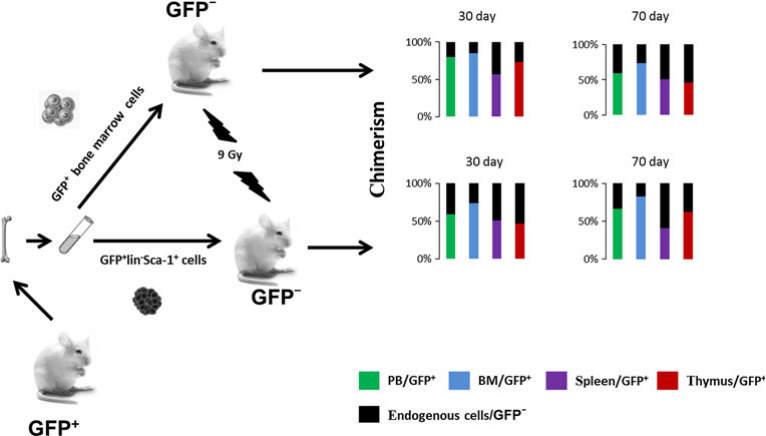
Model for the analysis of chimerism after cell transplantation. Our experimental model of C57BL/6-Tg(CAG-EGFP)C14-Y01-FM131Osb mice as donors of GFP^+^ bone marrow (BM) or GFP^+^lin^−^ Sca-1^+^ cells and C57Bl6/J mice as recipients (GFP^−^). Recipient animals were exposed to 9 Gy whole-body irradiation. Suspensions of GFP^+^ bone marrow cells (5 × 10^6^/ml) or GFP^+^lin^−^Sca-1^+^ cells (3 × 10^4^/ml) were transplanted by i.v. injection into recipient animals 3 hrs after irradiation. Flow cytometry analysis (FCA) of all GFP^+^ cells (%) was performed on days 30 and 70 after cell transplantation. We detected chimerism GFP^+^ cells (%)/GFP^−^ cells (%) in the peripheral blood (PB), bone marrow (BM), spleen and thymus.

In the present study, we describe a model using whole-body irradiation and the transplantation of bone marrow and HSCs (Fig. 1). We analysed the repair of haematopoiesis, the presence of extramedullary haematopoiesis and the circulation of GFP^+^-transplanted cells into non-haematopoietic tissues. Our results demonstrate that doses of whole-body irradiation also induced myeloablation and chimerism in tissues, and induced the entry of transplanted cells into the small intestine and liver.

## Materials and methods

### Animals

C57BL/6-Tg(CAG-EGFP)C14-Y01-FM131Osb mice (GFP^+^), obtained from Riken Laboratories (Tsukuba, Japan), were used as donors. C57Bl6/J mice (GFP^−^; Animal House, Medical Faculty Masaryk University, Brno, Czech Republic) were used as recipients. All studies were approved by the Charles University of Prague Medical Faculty, Hradec Králové-Czech Republic, and by the Institutional Animal Care and Use Committee.

### Drugs and solutions

Unless otherwise indicated, all standard chemicals and reagents were obtained from Sigma-Aldrich (St. Louis, MO, USA).

### GFP^+^ cell suspensions

Single cell suspensions were prepared individually from the bone marrow of the left femurs of all mice analysed (*n* = 6) in PBS containing 2% foetal calf serum (FCS). Whole heparinized peripheral blood and bone marrow cells were analysed by using a CyAN-ADP flow cytometer (DakoCytomation, Glostrup, Denmark).

### Sorting of lin^−^Sca-1^+^ (GFP^+^) bone marrow cells

**Fig. 2:**
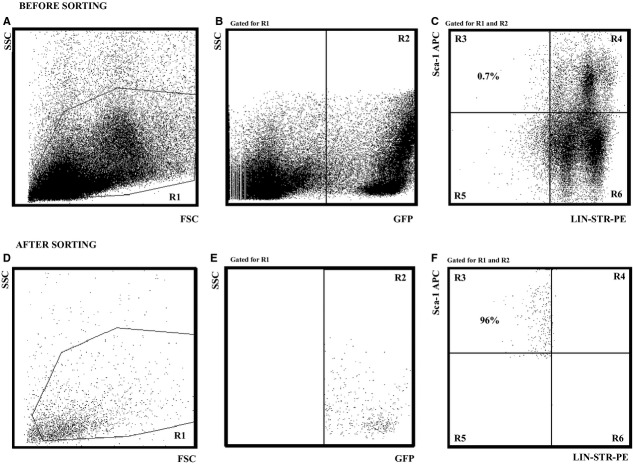
Isolation of lin^−^ Sca-1^+^ cells by FACS. The cell sorting was carried out on a FACS ARIA II cell sorter (Becton Dickinson). Before sorting, a bone marrow cell suspension (5 × 10^6^/ml) isolated from green fluorescent protein (GFP) mice was sorted for the presence of the GFP protein or incubated with 40 μl of biotin mouse Lineage Depletion Cocktail (BD IMAg™) and 5 μl rat antimouse Ly-6A/E(Sca-1)-APC (clone D7; Southern Biotech) for 30 min. at 4°C. Then, the cells were washed twice in Iscove*s modified Dulbecco*s Medium (IMDM) and stained with 5 μl of PE Streptavidin (BD Pharmingen; 15 min., 4°C). The cells were then washed twice in IMDM medium. The sorting gates were set (Fig. 1A–C), and sorted GFP^+^lin^−^Sca-1^+^ cells were collected in a tube containing IMDM medium with 2% FCS. (**A**) before sorting bone marrow cells by size (SSC) and granularity (FSC); (**B**) cell sorting and selection of GFP^+^ cells (quadrant R2); (**C**) the selected cells GFP^+^ lin^−^ (lin-Str-PE) Sca-1^+^ (Sca-APC) for applications; selected cells represent about 0.7% of GFP^+^ cells in the bone marrow. After sorting, an aliquot of the sorted cells was run on the FACS ARIA II to check the purity of the cell population (Fig. 1D–F); (**D**) cell profile after sorting; virtually all cells are small with minimal granularity; (**E**) all cells are GFP^+^; and (**F**) the final product is 96% sorted GFP^+^lin^−^Sca-1^+^ cells.

Sorting was carried out on an FACS ARIA II cell sorter (Becton Dickinson, Franklin Lakes, NJ, USA). Before sorting, bone marrow cell suspensions of 5 × 10^6^ cells/ml that were isolated from GFP mice were sorted for the presence of the GFP protein or incubated with 40 μl of biotin mouse Lineage Depletion Cocktail (BD IMAg™; Becton Dickinson) and 5 μl of rat anti-mouse Ly-6A/E(Sca-1)-APC (clone D7; Southern Biotech, Birmingham, AL, USA ) for 30 min. in a refrigerator. Then, the cells were washed twice in Iscove*s modified Dulbecco*s Medium (IMDM; Invitrogen) and stained with 5 μl of PE Streptavidin (BD Pharmingen, Heidelberg, Germany) for 15 min. at 4°C. Subsequently, the cells were washed twice in IMDM. The sorting gates were set to sort the cells. Sorted GFP^+^lin^−^Sca-1^+^ cells were collected in a tube containing IMDM with 2% FCS. After sorting, an aliquot of the sorted cells was run on the FACS ARIA II to check the purity of the cell population (Fig. 2).

### Irradiation and reconstitution

Recipient animals were exposed to 9 Gy whole-body irradiation from a ^60^Cobalt source (Chisotron, Chirana) at a dose rate of 1.3 Gy/min. Suspensions of bone marrow GFP^+^ cells (5 × 10^6^ cells/ml) or GFP^+^lin^−^Sca-1^+^ cells (3 × 10^4^ cells/ml) were transplanted by i.v. injection into recipient (GFP^−^) animals 3 hrs after irradiation.

### Identification of GFP^+^ cells and lineage phenotype-negative cells to determine cell chimerism in the peripheral blood, bone marrow, spleen and thymus

Single cell suspensions obtained from the bone marrow, spleen and peripheral blood were centrifuged, and the cell pellets were resuspended and incubated for 10 min. in EasyLyse solution (Dako, Glostrup, Denmark) to remove the red cells. The remaining cells were centrifuged, the pellets were resuspended and washed twice in ice-cold washing and staining buffer (PBS) containing 0.2% gelatin from cold water fish skin and 0.1% sodium azide, and the cell density was adjusted to 5 × 10^6^ cells/ml.

### Flow cytometry analysis

A total of 100 μl of cell suspension, equivalent to 5 × 10^5^ cells, was incubated with 5 μl of APC Mouse Lineage Antibody Cocktail (BD Pharmingen) for 30 min. on ice. Then, the cells were washed twice in ice-cold PBS, and the relative proportion of GFP^+^lin^−^Sca-1^+^ cells was determined on a nine-colour flow cytometer CyAn (Dako). Propidium iodide (PI) was added at a final concentration of 0.1 μg/ml immediately prior to acquisition. Acquisition and analysis were performed with Summit software (Dako). The detector sensitivity and off-line compensation of FITC and APC emission spectra overlaps were set by using unstained and single-colour stained samples and automatic compensation software utility.

### Detection of GFP^+^ cells in recipient tissue

Animals were killed 30 and 70 days after transplantation with GFP^+^ or GFP^+^lin^−^Sca-1^+^ cells. Each group comprised six mice and the tissue samples from these animals were processed for histology/cryosections. Each series contained at least 15 sections and these sections were processed for estimation of GFP^+^ cells, immunofluorescence and histochemical analysis. The left lobe of the liver and the duodenum were removed from recipient mice and fixed in 2% paraformaldehyde overnight. The next day, the tissues were first exposed to 10% sucrose and then to 20% sucrose before being left in 30% sucrose overnight. The tissues were frozen and cut into 10-μm-thick cryosections that were used for immunofluorescence. For fluorescence examination, the cell nuclei were counterstained with 4′,6-diamidino-2-phenylindole (DAPI), mounted in polyvinylalcohol/glycerol with 1,4-diazobicyclo-[2.2.2]-octane (DABCO), and at least three sections per sample were examined by using an Olympus (Prague, Czech Republic) BX51 microscope equipped with epifluorescence and a DP-71 camera.

For detection of antigens, briefly, the tissue sections were incubated with the primary antibodies goat antimouse albumin (Bethyl Laboratories Inc., Montgomery, TX, USA) and rat antimouse CD31 (BioLegend, San Diego, CA, USA) for 1 hr. The antigen-binding sites were identified after washing with PBS by using a corresponding biotinylated secondary anti-immunoglobulin that was visualized with streptavidin-Cy3. To identify pancytokeratin, mouse anti-cytokeratin AE1/AE3 was first coupled to antimouse immunoglobulin conjugated with Cy3 *in vitro*; for immunostaining, these complexes diluted in PBS with 0.1% Triton X-100 were used to avoid background staining. Histochemical detection of lipid droplets was performed with Oil red, and the activity of intestinal alkaline phosphatase was detected with BCIP/NBT (5-bromo-4-chloro-3-indolylphosphate/nitroblue tetrazolium) using incubation in 0.2 M Tris-HCl (pH 9.5) supplemented with 10 mM MgCl_2_ for 15 min.

### Analysis of GFP DNA content by qPCR

Quantitative PCR analysis was performed on a 7500 Fast Real-Time PCR System (Applied Biosystems, Foster City, CA, USA). Samples were collected from transplanted C57BL/6-Tg(CAG-EGFP)C14-Y01-FM131Osb (GFP^+^) mice. As positive controls, the corresponding tissues from GFP^+/+^ mice were used. The results obtained in mice were then expressed as the percentage of values of ΔCt measured in the respective tissues from GFP^+/+^ mice, which were considered to be 100% GFP (positive control). Respective tissues from non-transplanted C57Bl6/J mice served as the negative control, and qPCR analysis confirmed absence of GFP gene (data not shown). DNA was isolated from samples by using the DNeasy Tissue Kit and the QiaCube isolator (Qiagen Inc., Valencia, CA, USA). Quantitative PCR was performed in triplicate with 10 ng of DNA by using the TaqMan® Fast Universal PCR Master Mix (Applied Biosystems), an FAM-labelled probe and primer designed for GFP (EGFP Q1; Generi Biotech s.r.o, Hradec Kralove, Czech Republic) and the mouse housekeeping Polr2a gene (mPolr2a_G1; Generi Biotech s.r.o). Cycling conditions for both GFP and Polr2a products were 10 min. at 95°C, followed by 50 cycles of 95°C for 15 sec. and 60°C for 1 min. The relative DNA content was then calculated from the ΔCt of GFP and the housekeeping gene.

### Statistical analysis

**Fig. 3:**
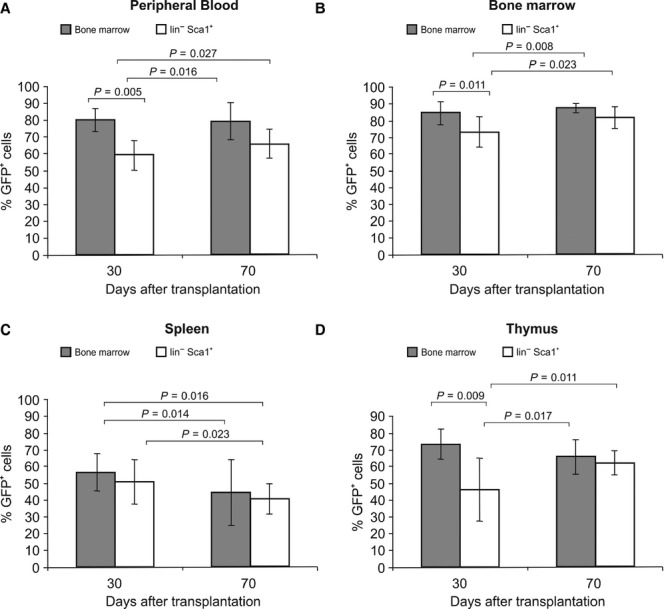
Migration of green fluorescent protein (GFP^+^) cells – levels of chimerism. Levels of chimerism were determined in the circulation after the transplantation of GFP^+^ bone marrow cells or GFP^+^lin^−^Sca-1^+^ cells on days 30 and 70 after transplantation: (**A**) peripheral blood, (**B**) bone marrow, (**C**) spleen and (**D**) thymus. Data are shown as the mean% ± SD. Data were evaluated using Student*s *t*-test (two-sided). The p values are given for each comparison. A value of *P* < 0.05 was considered statistically significant.

All data are expressed as mean ± SD. Data were evaluated by using the Student*s *t*-test (two-sided). The actual ‘p’ values are given for the data presented in Figures 3 and 6. A value of *P* < 0.05 or *P* < 0.01 was considered statistically significant.

## Results

### Haematopoietic recovery after transplantation

Haematopoietic recovery was assessed in lethally irradiated mice 30 and 70 days following transplantation with bone marrow GFP^+^ cells or GFP^+^lin^−^Sca-1^+^ cells separated from the bone marrow. The control group consisted of six normal mice without lethal irradiation. Evaluation of haematopoietic recovery after transplantation was based on the changes in lymphocyte, granulocyte, monocyte, erythrocyte and thrombocyte counts in the peripheral blood and the changes in colony-forming unit granulocyte-macrophage (CFU-GM) numbers of progenitor cells in the bone marrow (Table 1).

**Table 1:** Haematopoietic recovery after the transplantation of bone marrow or HSCs

	Peripheral blood					Bone marrow
		
	Lymphocyte (×10^9^/l)	Granulocyte (×10^9^/l)	Monocyte (×10^9^/l)	Erythrocyte (×10^9^/l)	Thrombocyte (×10^9^/l)	CFU-GM (colony/10^5^ MNC)
Control	4.02 ± 1.09	0.88 ± 0.35	0.18 ± 0.05	9.36 ± 2.63	618 ± 101	312.5 ± 26.2
30 day						
GFP^+^ BM	1.67 ± 0.94^*^	0.58 ± 0.37	0.19 ± 0.07	8.27 ± 0.63	437 ± 95^*^	98.2 ± 25.6^*^^*^
GFP^+^lin^-^Sca-1^+^	1.76 ± 0.74^*^	0.79 ± 0.91	0.22 ± 0.09	8.08 ± 1.02	647 ± 60	143.8 ± 19.5^*^
70 day						
GFP^+^ BM	7.21 ± 1.83	1.78 ± 0.57^*^^*^	0.31 ± 0.07^*^	8.82 ± 2.10	525 ± 87	275.2 ± 35.4
GFP^+^lin^-^Sca-1^+^	3.07 ± 0.75	0.58 ± 0.21	0.28 ± 0.11	9.89 ± 0.28	546 ± 46	226.5 ± 54.8

The table presents data showing significant differences in haematopoietic recovery 30 and 70 days after transplantation as indicated by the peripheral blood cell count and the total number of CFU-GM colonies in a control group (*n* = 6) and cells from GFP^+^ bone marrow (GFP^+^ BM; *n* = 6) or cells from bone marrow GFP^+^lin^−^Sca-1^+^ (*n* = 6). The differences between the control group and the BM group and between the control and GFP^+^lin^−^Sca-1^+^ groups were analysed, then the differences between the BM and GFP^+^lin^−^Sca-1^+^ groups were compared. The data are given as mean ± SD; CFU-GM (granulocyte-macrophage colony-forming units) colonies/10^5^ mononuclear cells (MNC). Data were evaluated by using Student*s *t*-test (two-sided). A value of *P* < 0.05 (^*^) or *P* < 0.01 (^*^^*^) when compared with the control group was considered statistically significant.

A significant decrease in the number of lymphocytes in the peripheral blood persisted 30 days after transplantation of the bone marrow cells and the separated cells, compared with the control group. In contrast to the control group, transplantation of full bone marrow resulted in a decrease in the number of platelets in the peripheral blood. This decrease, however, was not observed when separated cells were used for transplantation. By day 30, the number of granulocytes, monocytes and erythrocytes was normal. During evaluation, the restoration of haematopoiesis in the bone marrow was recorded after BMT. However, the CFU-GM number was significantly lower compared with the number in the control group. Conversely, transplantation of separated cells resulted in an increase in the CFU-GM number. Seventy days after the transplantation of bone marrow, a significantly higher number of cells and granulocytes were observed compared to the control group and the group transplanted with separated cells. An increase in the number of monocytes was observed after the transplantation of bone marrow in compared with the number of cells and granulocytes in the control group and the group transplanted with separated cells. The other parameters that were measured in both the peripheral blood and the bone marrow normalized 70 days after cell transplantation (Table 1).

### FACS analysis of GFP^+^ cells to assess chimerism in tissue after transplantation

Chimerism was assessed by the presence of GFP^+^ cells in the circulating peripheral blood, bone marrow, spleen and thymus 30 and 70 days following the transplantation of bone marrow GFP^+^ cells or GFP^+^lin^−^Sca-1^+^ cells separated from bone marrow into lethally irradiated recipient mice.

A significant difference in the number of circulating GFP^+^ cells in the peripheral blood was observed 30 and 70 days after the transplantation of GFP^+^ cells in the bone marrow. A similar result was observed after the transplantation of GFP^+^lin^−^Sca-1^+^ cells (Fig. 3A). In the peripheral blood, an increase in chimerism was observed, especially after the transplantation of GFP^+^lin^−^Sca-1^+^ cells. The share of the recipient*s own cells (GFP^−^) was greater than 40% at 30 days after transplantation and greater than 34% at 70 days after transplantation. The number of circulating recipient cells was significantly lower 30 and 70 days after BMT and did not exceed 20%. A significant difference in the percentage of circulating GFP^+^ cells was observed in the bone marrow 30 and 70 days after the transplantation of GFP^+^ cells. A difference was also recorded 30 and 70 days after the transplantation of GFP^+^lin^−^Sca-1^+^ cells. Another significant difference was observed when comparing bone marrow transplanted with GFP^+^lin^−^Sca-1^+^ cells after 30 and 70 days (Fig. 3B). An increase in chimerism in the bone marrow was observed 30 days after transplantation, especially after the transplantation of GFP^+^lin^−^Sca-1^+^ cells. In these mice, the percentage of the recipient*s own cells (GFP^−^) was greater than 27%. Seventy days after the transplantation of full bone marrow, the percentage of the recipient*s own cells was less than 12%, and after the transplantation of GFP^+^lin^−^Sca-1^+^ cells, the proportion of the recipients* bone marrow cells was less than 18%. Thirty days after transplantation of either bone marrow or lin^−^Sca-1^+^ cells, the spleens of recipient mice contained significantly different numbers of circulating GFP^+^ cells compared with 70 days after transplantation (Fig. 3C). Thirty days after transplantation, chimerism was observed in the spleens of recipient mice, with mice transplanted with bone marrow showing greater than 44% of the recipient*s own cells (GFP^−^); after 70 days, the spleens of mice transplanted with GFP^+^lin^−^Sca-1^+^ cells showed more than 59% of the recipient*s own cells (GFP^−^). The thymus of recipient mice showed a significant difference in the number of GFP^+^ circulating cells 30 days after transplantation of GFP^+^ bone marrow cells and GFP^+^lin^−^Sca-1^+^ cells compared with 70 days after transplantation (Fig. 3D). After 30 days, recipient mice transplanted with bone marrow showed increased thymic chimerism, with greater than 27% of cells derived from the recipient. Similarly, the recipient mice transplanted with GFP^+^lin^−^Sca-1^+^ cells showed thymic chimerism greater than 54% at 30 days after transplant and greater than 35% at 70 days after transplantation.

### Histology analysis of GFP^+^ cells in the small intestine after transplantation

**Fig. 4:**
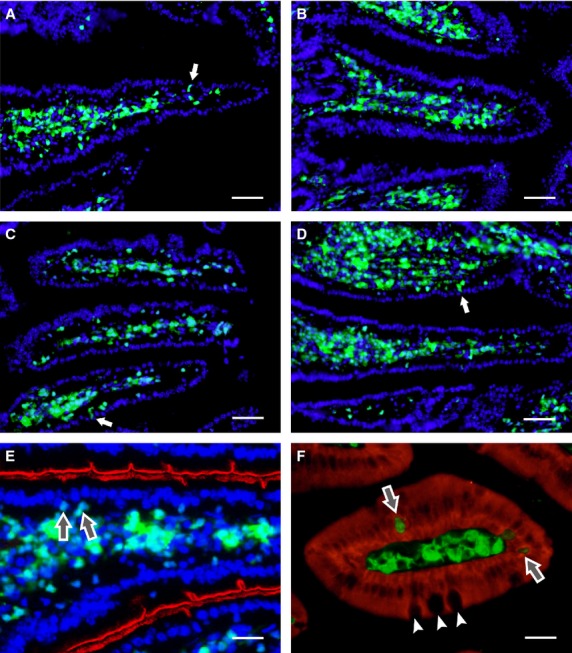
Histological analysis of cell engraftment in the small intestine. By day 30 after transplantation, the stroma of intestinal villi are richly colonized with green fluorescent protein (GFP^+^) cells derived from grafted bone marrow cells (**A**) or lin^−^ cells (**B**). GFP^+^ cells remain in the core of the villi for at least 70 days after the transplantation of unseparated bone marrow cells (**C** and **E**) or lin^−^ cells (**D**). A few GFP^+^ cells are closely associated with, or incorporated into, the epithelial lining (arrows). The brush border of enterocytes is indicated by red pseudocolour derived from the histochemical activity of intestinal alkaline phosphatase (**E**). Enterocytes express pancytokeratin (red), while GFP^+^ cells, including those incorporated in the epithelial lining (arrows), do not. Goblet cells (small arrows) are GFP^−^ (**F**). Cell nuclei were counterstained with DAPI; scale bar: A–D 100 μm, E 25 μm, F 20 μm.

The distribution of GFP^+^ transplanted cells was analysed microscopically in the small intestine and the liver of the recipient mice. By day 30, the duodenum of animals transplanted with full BM contained some GFP^+^ cells. A few transplanted cells were localized in the lamina propria of the intestinal villi, the connective tissue outside the intestinal crypts and the submucosa (Fig. 4A). Occasionally, GFP^+^ cells were found in the lining epithelium. However, the GFP^+^ nuclei of entrapped cells differed from enterocytes in size and shape. Only a few, sporadic cells were observed in the tunica muscularis externa and the adventitia. The small intestine of mice transplanted with sorted GFP^+^lin^−^Sca-1^+^ cells also contained a small amount of GFP^+^ cells that were distributed as single cells or clusters in the layers containing connective tissue, mostly in the lamina propria and the adjacent submucosa. These cells rarely entered the epithelial lamina. In the intestinal villi, the transplanted cells were localized mainly to the middle part of the villi (Fig. 4B). Seventy days after transplantation, the number of transplanted cells present in the small intestine increased. In the small intestine of mice transplanted with full bone marrow cells, a vast majority of the GFP^+^ cells were localized to the core of the villi, where the cells occupied the loose connective tissue. Microscopic examination confirmed that these cells were highly heterogeneous and differed in size, shape and cell nucleus morphology. Only a small percentage of GFP^+^ cells were found in the epithelial layer in the upper and middle part of the villi. However, the nuclei of such GFP^+^ intraepithelial cells were mostly located below the layer of the enterocyte nuclei. Observation by phase contrast showed that, in contrast to absorptive cells, the apical surfaces of GFP^+^ cells did not develop brush borders. Rare GFP^+^ cells were noticed on the top of the intestinal villi before being shed into the intestinal lumen (Fig. 4C). Numerous GFP^+^ cells were also present in the duodenum of mice transplanted with sorted lin^−^Sca-1^+^ cells, and these were mainly situated in the lamina propria and the submucosa. Fluorescent cells were rarely seen in the muscularis externa and the serosa (Fig. 4D). In the core of intestinal villi, GFP^+^ cells occupied both the central and peripheral parts, with numerous positive cells in contact with the basal lamina of the covering epithelium. The epithelium contained only rare, single GFP^+^ cells, but at a lower frequency than in mice grafted with full BM (Fig. 4E and F).

### Histology analysis of GFP^+^ cells in the liver after transplantation

**Fig. 5:**
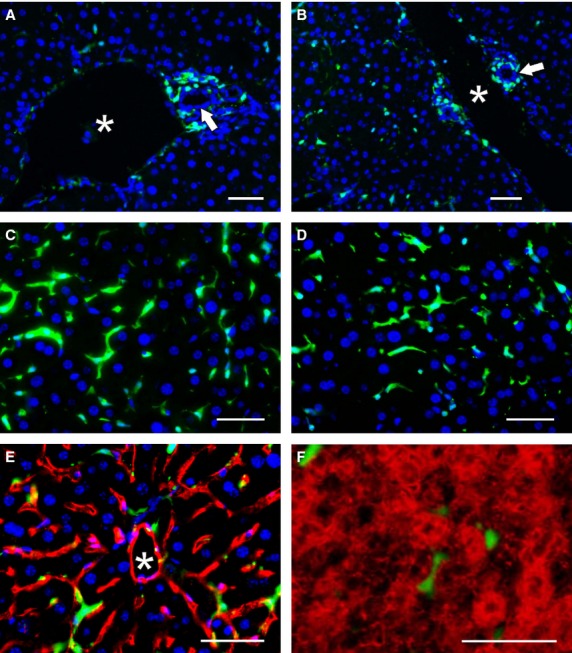
Histological analysis of cell engraftment in the liver. Thirty after transplantation, the stroma of the periportal area adjacent to the biliary ducts (arrows) and the interlobular vein (asterisk) contained green fluorescent protein (GFP^+^) cells derived from grafted bone marrow cells (**A**) or lin^−^ cells (**B**). Seventy days after grafting, GFP^+^ cells within the hepatic lobules differentiated into Kupffer-like-cells with stellate morphology (unseparated bone marrow cells – **C**, **E**; lin^−^ cells – **D**, **F**). GFP^+^ Kupffer-like-cells were closely apposed to CD31^+^ (red) endothelial cells lining the liver sinusoids; the central vein is indicated by an asterisk (**E**). Albumin^+^ hepatocytes (red) are morphologically distinct from the GFP^+^ Kupffer-like cells found in the perisinusoidal spaces (**F**). Cell nuclei were counterstained with DAPI; scale bar 100 μm.

Following transplantation, GFP^+^ cells were also present in the liver of recipients. Thirty days following transplantation with full bone marrow, many GFP^+^ cells were localized between hepatocyte plates. These GFP^+^ cells had elongated cytoplasmic processes and a single oval cell nucleus, which was smaller than that of hepatocytes. Many GFP^+^ cells accumulated in the perivascular spaces of the interlobular veins or in the connective tissue of the periportal areas (Fig. 5A). However, these cells were morphologically different as they had no cytoplasmic outgrowths. The liver parenchyma of mice that were transplanted with lin^−^Sca-1^+^ cells contained fewer GFP^+^ cells (Fig. 5B). Single GFP^+^ cells were usually located in the central areas of the liver lobules. The cells had cytoplasmic processes resembling the morphology of Kupffer-like-cells and were found in close vicinity to the endothelium of the liver sinusoids (Fig. 5C and E). Staining with oil red revealed that, in contrast to fat-storing cells and hepatocytes, the GFP^+^ cells did not accumulate lipid droplets. In contrast to hepatocytes, GFP^+^ cells never co-expressed albumin (Fig. 5F). Relatively frequently, GFP^+^ cells were also observed in the perivascular spaces of the interlobular blood vessels. Seventy days after transplantation with full bone marrow, the livers of the transplanted mice contained GFP^+^ Kupffer-like-cells in the liver lobules and in the periportal areas, mainly around the interlobular blood vessels. Occasionally, these cells were present in small groups in the vicinity of the interlobular veins. In the mice transplanted with lin^−^Sca-1^+^ cells, the GFP^+^ cells had the same morphology and distribution (Fig. 5D and F). Specifically, GFP^+^ Kupffer-like-cells were found between plates of hepatocytes and other GFP^+^ cells aggregated in the periportal spaces. GFP^+^ cells were rarely seen in the walls of the interlobular veins or arteries. In rare cases, the interlobular biliary ducts were surrounded by GFP^+^ cells (Fig. 5A and B).

### qRT-PCR analysis of GFP^+^ cells in tissues after transplantation

**Fig. 6:**
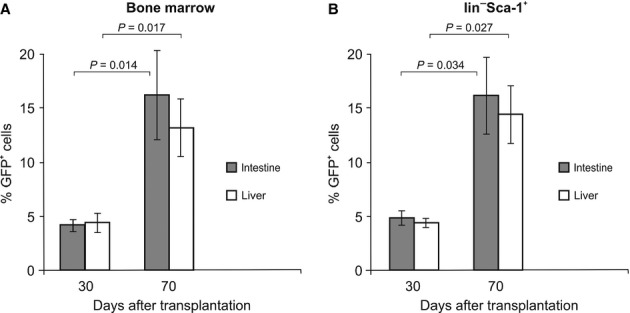
qPCR detection of green fluorescent protein (*GFP*) DNA in recipient tissues. Expression of the *GFP* gene on day 30 and 70 after the transplantation of bone marrow cells or HSCs into lethally irradiated mice. Transplanted cells carrying the *GFP* gene were detected in the recipient tissues by quantitative PCR. The highest values were reached by day 70 after the transplantation of bone marrow cells or HSCs – the small intestine and liver in comparison with day 30. Data are the mean% ± SD. Statistical significance was determined using Student*s *t*-test. The p values are given for each comparison. The differences were considered significant at *P* < 0.05.

To determine the extent of participation of lin^−^Sca-1^+^ cells in haematopoietic repair and their distribution in recipient tissues, we carried out qPCR analysis of selected non-haematopoietic tissues from both experimental groups to identify the presence of the *GFP* gene that was used as an endogenous marker of transplanted cells. As qPCR data are limited in terms of distinguishing cell migration and proliferation, all tissue samples were processed in parallel for histochemistry (see above). Seventy days after transplantation, the group that was transplanted with full bone marrow showed a significant increase in GFP^+^ cells in the liver and the small intestine. Similar results were also observed after the transplantation of sorted GFP^+^lin^−^Sca-1^+^ cells, with an increase in GFP^+^ cells observed in the liver and the small intestine. Thirty and 70 days after transplantation, there was a significant difference in the number of GFP^+^ cells migrating into the liver and intestine between the two experimental groups of mice (Fig. 6).

## Discussion

Herein, we report that a lethal dose of whole-body irradiation has limited effects on the migration and homing of bone marrow and HSCs (Lin^−^Sca-1^+^ cells) after their transplantation.

Limited damage was found in all regions with regenerative processes. Haematopoietic stem cells are self-renewing cells of the bone marrow that are responsible for maintaining homeostasis through the continuous production of all cellular blood components, including leucocytes, erythrocytes, lymphocytes and platelets. Despite performing vital functions, HSCs themselves are relatively rare cells. It is estimated that in the murine bone marrow, only ∼0.01% of nucleated cells, or ∼5000 cells per mouse, possess the profile of HSCs [13]. Haematopoietic stem cells are typically isolated based on surface marker expression, with the most common defining population being c-kit^+^Sca-1^+^Lin^−^ cells [14]. This rarity poses a significant barrier to many experimental techniques and has proved particularly troublesome when investigating the mechanisms of extramedullary HSC homing *in vivo*.

In our earlier studies, we monitored their plasticity, migration and reparative potential [7,11,12,15]. In particular, transplanted bone marrow cells represent a heterogeneous system, and their plasticity allows the regeneration and repair of damaged tissues and the maintenance of homeostasis in the organism [7,16]. This repair model applies to a number of situations, for example, the treatment of ARS by using full bone marrow or HSCs separated from bone marrow [9,11], neural SCs [5,7] or mesenchymal SCs [17].

Of interest, it was reported that the GFP^+^Lin^−^Sca^−^1^+^ cell population possesses the potential to differentiate into mesenchymal origin cells [18]. The cotransplantation of MSCs with haematopoietic stem and progenitor cells improved donor engraftment and enhanced self-renewal [19,20], suggesting the appropriateness of the use of murine Lin^−^Sca^−^1^+^c-Kit^+^ cells or CD34lo/^−^Sca^−^1^+^Thy1.1^+^/loCD38^+^c-Kit^+^Lin^−^ cells. However, others have demonstrated that Lin^−^Sca^−^1^+^ cells proliferate in the BM and subsequently mobilize in the peripheral blood in response to liver injury by CCl(4) or an injury simulating condition [21]. Therefore, it is known that HSCs presenting the cell surface markers Lin^−^Sca^−^1^+^ have the potential to differentiate into MSCs. In our hands and in those of other groups, Lin^−^Sca^−^1^+^c-kit^+^ cells present a number of advantages for their use [22,23].

All of the activities of HSCs in mice are related to lineage- (lin^-^), Sca-1^+^ and CD117^+^ (c-kit^+^) cells [24]. Randall *et al*. showed that a population of adult bone marrow that was characterized as lin^-^CD117^+^Sca-1^+^ contained most of the day-12 (CFU-S) [25]. However, this population contained at least two subpopulations with different function: (*i*) a subpopulation consisting of cells with high self-renewal capacity of long-term repopulating LT-HSCs, and (*ii*) a subpopulation consisting of cells more committed to the short-term repopulation of a particular lineage ST-HSCs. The mouse has very few HSC-LT-HSC in the bone marrow (one to two cells per 100,000 bone marrow cells). It was determined that the functional population of LT-HSC corresponds with the phenotype lin^−^Sca-1^+^CD117^+^CD38^+^CD34^−^, and such cells can be isolated by using five-colour flow cytometry and sorting [26]. However, these cells, when transplanted alone into lethally irradiated recipients, were not able to reconstitute the functions of the haematopoietic system quickly enough to prevent death. After the transplantation of lin^−^Sca-1^+^CD117^+^CD38^+^CD34^−^ cells together with lin^−^Sca-1^+^CD117^+^CD38^+^CD34^+^ cells (ST-HSC), haematopoiesis was restored quickly and sufficiently for long-term survival. Not only are LT-HSC rare in the bone marrow, but it was also found that only 20% of them are involved in the long-term reconstitution of haematopoiesis, suggesting that subpopulations of LT-HSC with different functions may also exist [27,28]. During the maturation of cells, the CD38 antigen seems to be lost at the same time as CD34 begins to be expressed, as the majority of CD38^+^ cells are also CD34^−^, and the majority of CD38^−^ cells are CD34^+^ [26].

More abundant HSCs in the mouse are the ST-HSC. This cell phenotype was described by Yang *et al*. [24], who studied lin^−^Sca1^+^CD117^+^ cells and found that the vast majority of them are CD34^+^. These lin^−^Sca-1^+^CD117^+^CD34^+^ cells have the ability to form CFU-S and are responsible for the quick reconstitution of haematopoiesis when transplanted to lethally irradiated recipients, but they cannot replenish undifferentiated LT-HSC with high self-renewal capacity. Therefore, we believe that for clinical purposes one could use only those cells that have the surface markers Lin^−^Sca-1^+^. It is obvious that these cells have the potential to differentiate into mesenchymal cells, and our experimental results (both present and previous) proved that the reparative potential of these cells is very high. We therefore focused on testing this population of HSCs with more possibilities for their use in the treatment of damaged tissues.

Experiments and clinical observations have revealed a few important factors that are directly related to the application of HSCs in the regeneration of damaged tissues. The first factor is the mobilization of these cells from the bone marrow and their circulation in the body. This behaviour is supported by a number of reports showing that the number of endogenous HSCs circulating in the peripheral blood increases after various injuries [12,29,30]. Another important factor is the circulation of these cells in the body and their homing to damaged tissues. *In vivo* studies have shown that the homing kinetics of HSCs and progenitor cells to the bone marrow is dependent on the rates at which these cells roll, form adhesions and transmigrate [31,32]. For example, GFP^+^ bone marrow–derived transplanted cells were detectable in the brain following 10 Gy whole-body irradiation. This result may have been as a result of the effects of irradiation on the blood–brain barrier or on the blood–cerebrospinal fluid barrier, resulting in the weakening of the junctions between cells, which are the basis of the permeability of these barriers. Alternatively, the release of chemoattractant factors, such as VEGF, could have induced the entry of the cells [33].

Previously, it was shown that cotransplantation with MSCs would improve the efficacy of the transplantation of gene-modified HSCs in primates, with enhanced engraftment in the BM as well as increased chimerism in the peripheral blood through migration and homing [19]. Quesenberry *et al*. have shown that the keys to high-level non-toxic chimerism in syngeneic models are stem cell toxicity, non-myelotoxic host treatment as provided by 100-cGy whole-body irradiation and relatively high levels of marrow SCs. This approach was successful in H-2 mismatched B6.SJL to BALB/c marrow transplants and other models [34–38] where a high-level allogeneic chimerism was achieved by total-body irradiation in the clinical application of BMT [39]. In addition, the potential of the BMT approach was successfully achieved in a mouse model including the use of virally transduced cells [40].

In our experiments, we have focused on the homing of transplanted cells derived from the bone marrow to non-haematopoietic tissues. As a starting model, we used genetically modified mice expressing GFP and isolated whole bone marrow or bone marrow cells that were further separated by using a cell sorter to obtain a GFP^+^lin^−^Sca-1^+^ HSC population. These two populations of cells were transplanted into GFP^−^ recipients. The recipients were exposed to a lethal dose of 9 Gy LD total body irradiation, and 3 hrs after irradiation, they were transplanted with GFP^+^ cells. Analysis of the kinetics and regenerative potential of the transplanted GFP^+^ cells was performed 30 and 70 days after cell transplantation. The transplanted cells from the bone marrow colonized not only the bone marrow but also the non-haematopoietic tissues. The transplanted cells were also involved in extramedullary haematopoiesis [11,12]. Our analysis confirmed that the gastrointestinal tract, primarily the liver and intestine (non-haematopoietic system), was colonized by the transplanted cells. It was found that the systemic administration of HSC lines during hepatic ischaemia-reperfusion may favourably affect and maintain the homeostasis and function of the liver tissue [41]. Our experiments provided similar results using another model of damage, namely, total-body irradiation plus splenectomy [12]. In our study setting, whole-body irradiation was sufficient to induce a strong chimerism in the periphery, both in the circulation and in haematopoietic organs such as the spleen and thymus.

These results confirm that irradiation allows the entry of transplanted cells into the gastrointestinal tract, particularly the small intestine and liver. Grafted cells were found in the intestinal villi, and 30 days after transplantation, the stroma of the intestinal villi were richly colonized with GFP^+^ cells derived from grafted bone marrow cells or GFP^+^lin^−^Sca-1^+^ cells. GFP^+^ cells remained in the core of the villi for at least 70 days after the transplantation of unseparated bone marrow cells or GFP^+^lin^−^Sca-1^+^ cells. Thirty days after transplantation, the transplanted cells in the liver were distributed in the stroma of the periportal area adjacent to the biliary ducts and the interlobular vein. Seventy days after transplantation, the GFP^+^ cells within the hepatic lobules differentiated into Kupffer-like-cells with a stellate morphology. From these data, it can be assumed that after total body irradiation, which is associated with increased risk, the process of tissue repair was stimulated by cells from the recipient. However, transplanted donor cells can help the repair process by complementing the missing pool of cells needed for the maintenance of homeostasis. A limiting factor in the repair process is the degree of heterogeneity of the transplanted cells [33,42] and the ability of the microenvironment to repair the induced changes after oxidative damage [43].

## Conclusions

Taken together, our results document that transplanted bone marrow cells, which are a highly heterogeneous population, also showed repair potential as has been observed with a sorted, less heterogeneous population of cells (lin^−^ Sca-1^+^). Further evidence of the repair potential of transplanted bone marrow cells was also observed in the chimerism after the transplantation of GFP^+^lin^−^Sca-1^+^ cells, which significantly increased haematopoiesis, whereas the repair induced by endogenous cells was significantly reduced. Our results demonstrate that grafted bone marrow cells or GFP^+^lin^−^Sca-1^+^ cells are not transient in the gastrointestinal tract and thus could be used for the long-term treatment of various pathologies. The transplantation of these cells could also be a one-time treatment option if myeloablation-induced peripheral chimerism alone is not sufficient to induce the entry of transplanted cells into non-haematopoietic tissues.
